# Supplementary material for: Chromothripsis during telomere crisis is independent of NHEJ, and consistent with a replicative origin
Source: Genome Res. 2019 May;29(5):737–49. doi: 10.1101/gr.240705.118 (PMC6499312; doi:10.1101/gr.240705.118)
Supplement: Supplemental Material [file supp_gr.240705.118_Supplemental_file_1.zip › contigs/annotated_contigs/DB46/contig.2.DB46_length_381_mean_cov_4.72440944882.docx]

**DB46_length_381_mean_cov_4.72440944882**

```
TATTATTTTATAAGGATACATATATATATATATATATATATATATATATATATACTATATATAT|ATA|CTTTCCCATGCTATTCTCAT
 >chr14:86785504-86785567 + E=3e-22                                 >chr14:86786651-867
GATAGTGAATAAGTCTCAAAAGATCTGATGACTTCATAAAGGGGAGTTTCCCTGCACAAATTCTCTTCTTTTGTCTGCCACCATATGAG
86965 - E=6e-178
ACGTGCCTTTCACCTTGTGCCATGATTGTGAGGCCTCCCCAGCCAGGTGGACCATGAGTTCATTAAACCTCTTTCTTTTGTAAATTTCC

CAGTCTTGGGTATGTCTTTATCAGCATCCTGAAAACAGATTAATACACATTAATATGTCATTCAAATGAATGTAGGGTTAAGTTGTTCA

ATTCATTGGATAATTACCAACTCTATA
```
